# Presenting native-like HIV-1 envelope trimers on ferritin nanoparticles improves their immunogenicity

**DOI:** 10.1186/s12977-015-0210-4

**Published:** 2015-09-26

**Authors:** Kwinten Sliepen, Gabriel Ozorowski, Judith A. Burger, Thijs van Montfort, Melissa Stunnenberg, Celia LaBranche, David C. Montefiori, John P. Moore, Andrew B. Ward, Rogier W. Sanders

**Affiliations:** Department of Medical Microbiology, Academic Medical Center, University of Amsterdam, 1105 AZ Amsterdam, The Netherlands; Department of Integrative Structural and Computational Biology, IAVI Neutralizing Antibody Center, Collaboration for AIDS Vaccine Discovery (CAVD), Center for HIV/AIDS Vaccine Immunology and Immunogen Discovery, The Scripps Research Institute, La Jolla, CA 92037 USA; Department of Surgery, Duke University Medical Center, Durham, NC 27710 USA; Department of Microbiology and Immunology, Weill Medical College of Cornell University, New York, NY 10065 USA

**Keywords:** HIV-1, Envelope glycoprotein, Ferritin, Nanoparticles, Vaccine, SOSIP, BG505

## Abstract

**Background:**

Presenting vaccine antigens in particulate form can improve their immunogenicity by enhancing B cell activation.

**Findings:**

We describe ferritin-based protein nanoparticles that display multiple copies of native-like HIV-1 envelope glycoprotein trimers (BG505 SOSIP.664). Trimer-bearing nanoparticles were significantly more immunogenic than trimers in both mice and rabbits. Furthermore, rabbits immunized with the trimer-bearing nanoparticles induced significantly higher neutralizing antibody responses against most tier 1A viruses, and higher responses (but not significantly), to several tier 1B viruses and the autologous tier 2 virus than when the same trimers were delivered as soluble proteins.

**Conclusions:**

This or other nanoparticle designs may be practical ways to improve the immunogenicity of envelope glycoprotein trimers.

## Findings

An HIV-1 subunit vaccine should induce a broad and potent neutralizing antibody (NAb) response against the envelope glycoprotein spike (Env) [1]. Soluble, stable mimics of the native spike, such as the BG505 SOSIP.664 gp140 trimer, might be good starting points for such a vaccine [2–5]. These trimers bind virtually all known broadly neutralizing antibodies (bNAbs) but almost no non-neutralizing antibodies (non-NAbs), and adopt a native-like conformation with a well-defined structure [2, 6–8]. Furthermore, unlike other gp140 proteins, soluble, adjuvanted BG505 SOSIP.664 trimers induce NAbs against the autologous, neutralization-resistant (tier 2) virus efficiently in animals [9]. Licensed subunit vaccines against viral pathogens, such as hepatitis B virus and human papillomavirus, are however particulate antigens [10]. The greater size and the capacity for multivalent antigen presentation and B cell receptor cross-linking provide such particulate vaccines with advantages over soluble proteins for inducing antibody responses [11]. For example, fusing eight influenza hemagglutinin (HA) trimers or engineered HA stem antigens to *Helicobacter pylori* ferritin greatly improved NAb responses against influenza in animals [12, 13].

**Fig. 1:**
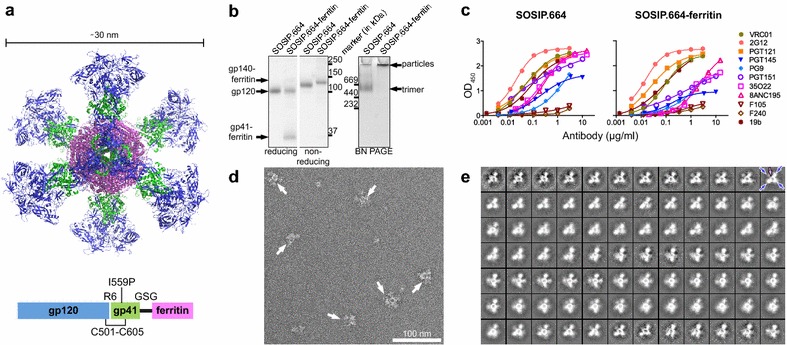
Design and biochemical characterization of BG505 SOSIP.664-ferritin nanoparticles. **a** *Top*: model of eight BG505 SOSIP.664 trimers (PDB: 4TVP) with gp120 subunits in *blue* and gp41 subunits in *green*, displayed on the *H. Pylori* ferritin nanoparticle (in *violet*, PDB: 3BVE), viewed down one of the threefold axes of the ferritin particle. The figure was drawn using Pymol [20]. *Bottom*: the BG505 SOSIP.664-gp140-ferritin construct. The hexa-arginine furin cleavage site (R6) [21], the SOS disulfide bond between gp120 and gp41 (C501–C605) [22], and the I559P substitution that facilitates trimerization [23] are indicated on the SOSIP.664 component, to which the ferritin moiety is linked via a Gly-Ser-Gly (GSG) spacer. **b** Coomassie-stained reducing and non-reducing SDS-PAGE (*left*) and BN-PAGE (*right*) gels comparing soluble SOSIP.664 trimers and SOSIP.664-ferritin nanoparticles. The nanoparticles were too large to enter BN-PAGE gels efficiently, but were visible at the top of the *lanes* (Fig. 1b, *right panel*, *right lane*). **c** Representative ELISA binding curves of a panel of antibodies to SOSIP.664 trimer (2.0 μg/ml) and SOSIP.664-ferritin (0.45 μg/ml) with 2G12 as loading control. **d** Unprocessed electron micrograph showing individual SOSIP.664-ferritin particles (indicated by *arrows*). Protein samples were prepared on carbon-coated copper grids. Imaging was carried out using an FEI Tecnai T12 microscope operating at 120 keV [2]. Images were collected using a Tietz TemCam-F416 CMOS camera at 1 µm defocus with an average dose of 25 electrons/Å^2^ and a magnification of ×52,000. **e** 84 NS-EM 2D class averages of SOSIP.664-ferritin particles. The SOSIP.664 spikes (*blue arrows*) and the ferritin cage (*magenta arrow*) are highlighted in the *top right* 2D class average image

Modeling showed that *H. Pylori* ferritin (GenBank accession no. NP_223316) could potentially present eight BG505 SOSIP.664 trimers. Therefore we fused the ferritin N-terminus, starting from Asp5, to the SOSIP.664 C-terminus, separated by a Gly-Ser-Gly (GSG) linker (Fig. 1a). The SOSIP.664-ferritin plasmid was co-transfected into 293F cells with a furin plasmid to maximize trimer cleavage and ensure it adopts a native conformation [14]. To select for antigenically and structurally well-folded Env proteins, the secreted nanoparticles and control trimers were purified using PGT145 bNAb-affinity chromatography [15]. Judged by BN-PAGE and SDS-PAGE analysis followed by Coomassie staining this purification method yielded highly pure (>95 % purity) SOSIP.664 trimer and SOSIP.664-ferritin protein preparations (Fig. 1b). SDS-PAGE also confirmed that the SOSIP.664 component of the nanoparticles was cleaved efficiently between gp120 and gp41 (Fig. 1b, left panel).

The antigenic structure of SOSIP.664 trimers and SOSIP.664-ferritin was compared using ELISA. Proteins were captured using *Galanthus nivalis* lectin and probed with bNAbs and non-NAbs (Fig. 1c). Several bNAbs that bind to distinct Env epitopes (VRC01, PGT121, PG9) showed similar binding to SOSIP.664 and SOSIP.664-ferritin, moreover non-NAbs (F105 and F240) displayed similarly poor reactivity with both proteins (Fig. 1c). We did observe lower affinity of gp120/gp41 interface (8ANC195, 35O22 and PGT151) and gp41 (3BC315) bNAbs for SOSIP.664-ferritin, which might be explained by steric hindrance of neighboring trimers on the nanoparticle (Fig. 1c).

The purified nanoparticles were analyzed by negative stain electron microscopy (NS-EM). More than 70 % of the particles on the EM grid resembled ferritin cages with protruding spikes that were 30–40 nm in diameter (Fig. 1d). When single particles were automatically picked and processed as described elsewhere [2], 2D class averages representing views along the three- and fourfold symmetry axes suggested that 65–80 % of the SOSIP.664-ferritin particles were fully decorated with Env trimers (three and four spikes visible, respectively) (Fig. 1e). The lack of views along the twofold symmetry axis (i.e. six spikes visible) may be a result of the immobilization on the EM grid or flexibility of the GSG-linker that affects the alignment of the particles and visualization of each Env trimer.

**Fig. 2:**
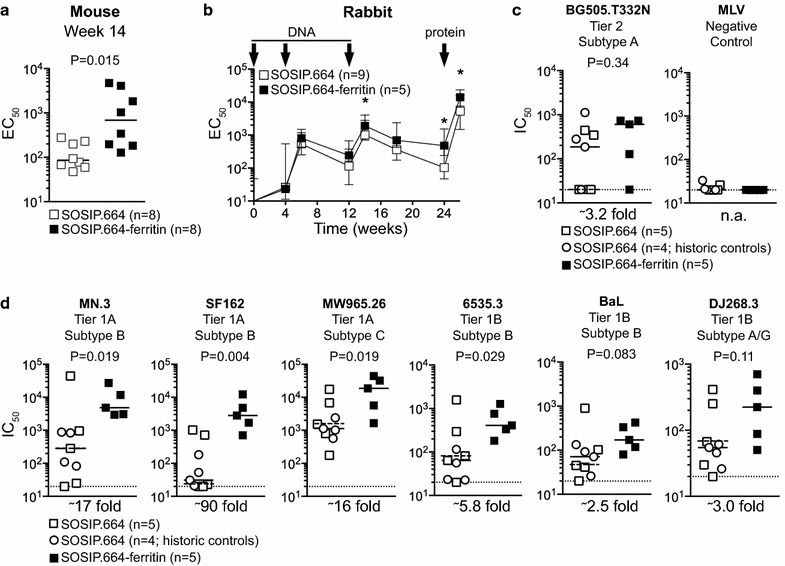
Induction of increased antibody responses by BG505 SOSIP.664-ferritin in mice and rabbits. **a** Eight BALB/C mice were immunized three times (at weeks 0, 4 and 12) with either 2.8 μg of BG505 SOSIP.664 trimer or BG505 SOSIP.664-ferritin protein formulated with 25 μg MPLA adjuvant. The midpoint binding (EC_50_) titers to BG505 SOSIP.664 trimer were determined at week 14 by Ni-NTA ELISA [2]; the median titers are denoted by horizontal lines. Statistical analysis was performed using a two-tailed Mann–Whitney U test. **b** Two groups of five New Zealand White rabbits received intramuscular immunizations at weeks 0, 4 and 12 with 200 μg of a non-adjuvanted DNA plasmid via electroporation of the quadriceps, followed by a protein boost at week 24 with 17 µg of protein in ISCOMATRIX™ adjuvant (75 units per rabbit) [24]. The DNA plasmids encoded either the soluble BG505 SOSIP.664 gp140 or the BG505 SOSIP.664 gp140-ferritin nanoparticles; none of the plasmids encoded furin. The protein boost was, correspondingly, either soluble SOSIP.664 trimers or SOSIP.664-ferritin particles, in both cases purified by a PGT145 bNAb column. The four historic control rabbits (indicated by *circles* in *panel* **b**) received identical DNA priming, but were then boosted with ISCOMATRIX™ adjuvanted (75 units per rabbit) soluble BG505 SOSIP.664 trimers (40 μg) that had been purified using 2G12-affinity chromatography followed by size exclusion chromatography (SEC) [2], which are antigenically identical to PGT145-purified BG505 SOSIP.664 trimers [25]. Anti-trimer serum binding titers over the course of the experiment were tested in D7324-capture ELISA using 2G12/SEC purified D7324-tagged BG505 SOSIP.664 trimers (0.5 μg/ml), essentially as described before [2, 9]. The medians of the midpoint binding titers (±error) are plotted. *Asterisks* indicate significant differences at specific time points (two-tailed Mann–Whitney U test; *P < 0.05). **c** Midpoint neutralization (IC_50_) titers against the autologous neutralization-resistant (*tier 2*) virus, BG505, and against the negative control, MLV, at week 26. **d** IC_50_ titers against a panel of heterologous neutralization-sensitive (*tier 1A* and *tier 1B*) viruses at week 26. The IC_50_ titers in **c** and **d** were determined using the TZM-bl neutralization assay. The pre-bleed samples lacked neutralization activity (not shown). Neutralization assays were performed either at the Academic Medical Center (SF162, 6535.3, ZM197M, HXB2, DJ268.3, BaL, ZM109F, 94UG103, 92RW020, Q23env17 and MLV) or the Duke University Medical Center (DUMC) (BG505.T332 N, MN.3, MW965.26, Q259.d2.17, Ce1176_A3, Q769.d22, Q842.d12, YU2, Q23env17 and MLV). The fold difference in median IC_50_ titer (*horizontal lines*) is depicted below the graphs. The *dotted horizontal lines* in the BG505 SOSIP.664 group represent the median titers for the five animals from the current experiment, i.e. excluding the four control sera. The titers were very similar when the four control sera were included or excluded. Statistical differences between the nine trimer-immunized rabbits and the five nanoparticle-immunized rabbits were determined using a two-tailed Mann–Whitney U test

We first immunized mice (approved by the AMC animal ethics committee: DMB-102836; n = 8 mice per group) to compare the antibody response of SOSIP.664-ferritin nanoparticles with soluble (i.e. monovalent) SOSIP.664 trimers. The anti-trimer binding responses were eightfold higher in mice vaccinated with nanoparticle-displayed trimers compared to soluble trimers (medians: 86 vs. 686; P = 0.015) (Fig. 2a). We next immunized rabbits (approved by the Covance Institutional Animal Care and Use Committee (IACUC): 0082-14; n = 5 rabbits per group), using a triple DNA-prime, protein-boost regimen (Fig. 2b). Given the limited group sizes and the large spread in neutralization titers generally observed in other HIV-1 vaccination studies [9], we included historic control sera from four rabbits to increase the statistical power of this study. These rabbits were immunized with the soluble trimers in an independent experiment using the same DNA prime + protein boost protocol (approved by the Covance IACUC: 0001-14; n = 4 rabbits per group; unpublished results). As expected, the anti-trimer binding antibody responses rose and fell between immunizations, and were boosted by the protein-only immunization [9, 16]. The titers were two- to threefold higher at several time points for the rabbits given SOSIP.664-ferritin nanoparticles compared to the soluble trimers. Although the improved immunogenicity was less pronounced in rabbits compared to mice, it is consistent with other observations showing the benefits of particulate antigen presentation [12, 17, 18] (Fig. 2b).

We used the TZM-bl cell neutralization assay and viruses from different clades to assess the serum NAb titers 2 weeks after the protein boost in rabbits [19]. Sera from 4/5 rabbits given the SOSIP.664-ferritin nanoparticles neutralized the autologous BG505.T332 N tier 2 virus, and the median titer in this group was higher than in the soluble trimer group (603 vs. 186). However, because of the small group sizes, the difference was not statistically significant (P = 0.34) (Fig. 2c). The NAb titers against heterologous tier 1 viruses were also higher in the rabbits that received SOSIP.664-ferritin nanoparticles (Fig. 2d). Median NAb titers against tier 1A viruses were 10- to 90-fold higher in the nanoparticle group: MN.3 (4,857 vs. 282; P = 0.019); SF162 (2,799 vs. 31; P = 0.004); MW.965 (18,563 vs. 1,127; P = 0.019). For the more resistant tier 1B viruses the titers were also higher, although this did not reach statistical significance in all cases: 6535.3 (472 vs. 82; P = 0.029); BaL (171 vs. 71; P = 0.083); DJ286.3 (195 vs. 64; P = 0.11). The tier 1B viruses HxB2, Q23env17, ZM109F and ZM197M and the tier 2 viruses 94UG103, 92RW020, Q259.d2.17, Q769.d22, Q842.d12 (all clade A), YU2 (clade B) and Ce1176_A3 (clade C) were not neutralized by any rabbit sera (data not shown).

## Conclusions

We conclude from this exploratory study that the nanoparticle display of SOSIP.664 trimers improves the magnitude of the overall antibody response and neutralization breadth at the tier 1 level. We are seeking to solve the substantial problem of inducing a bNAb response (at the tier 2 level) by improving the design of native-like trimers such as BG505 SOSIP.664 and/or how they are used as immunogens. If and when this goal is achieved, the superior immunogenicity of a particulate antigen presentation should be valuable.
